# (*E*)-*N*-(3,4-Dimethoxy­pheneth­yl)-3-methoxy­but-2-enamide

**DOI:** 10.1107/S1600536810001972

**Published:** 2010-01-23

**Authors:** Xiang Li

**Affiliations:** a Chemistry and Chemical Engineering Department, Henan University of Urban Construction, Pingdingshan 467044, People’s Republic of China

## Abstract

In the crystal of the title compound, C_15_H_21_NO_4_, inter­molecular N—H⋯O hydrogen bonds link mol­ecules related by translation along the *c* axis into hydrogen-bonded chains. C—H⋯O links are also present. The dihedral angle between benzene ring and enamide group is 50.08 (3)°

## Related literature

For the applications of the title compound, see: Bernhard & Snieckus (1971 ▶); Ma *et al.* (2006 ▶). For bond-length data, see Allen *et al.* (1987 ▶).
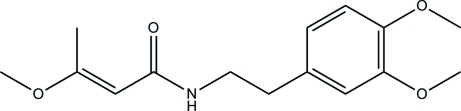


## Experimental

### 

#### Crystal data

C_15_H_21_NO_4_
*M* *_r_* = 279.33
Monoclinic, 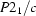

*a* = 12.509 (3) Å
*b* = 14.930 (3) Å
*c* = 8.2998 (17) Å
β = 107.59 (3)°
*V* = 1477.5 (5) Å^3^
*Z* = 4
Mo *K*α radiation
μ = 0.09 mm^−1^
*T* = 173 K
0.30 × 0.20 × 0.15 mm

#### Data collection

Rigaku Mercury CCD/AFC diffractometer
Absorption correction: multi-scan (*CrystalClear*; Rigaku, 2007 ▶) *T*_min_ = 0.973, *T*_max_ = 0.987
10754 measured reflections
2591 independent reflections
2435 reflections with *I* > 2σ(*I*)
*R*_int_ = 0.049

#### Refinement

*R*[*F* ^2^ > 2σ(*F* ^2^)] = 0.059
*wR*(*F* ^2^) = 0.133
*S* = 1.18
2591 reflections
181 parameters
H-atom parameters constrained
Δρ_max_ = 0.16 e Å^−3^
Δρ_min_ = −0.18 e Å^−3^

### 

Data collection: *CrystalClear* (Rigaku, 2007 ▶); cell refinement: *CrystalClear*; data reduction: *CrystalClear*; program(s) used to solve structure: *SHELXS97* (Sheldrick, 2008 ▶); program(s) used to refine structure: *SHELXL97* (Sheldrick, 2008 ▶); molecular graphics: *SHELXTL* (Sheldrick, 2008 ▶); software used to prepare material for publication: *SHELXTL*.

## Supplementary Material

Crystal structure: contains datablocks I, global. DOI: 10.1107/S1600536810001972/hg2630sup1.cif

Structure factors: contains datablocks I. DOI: 10.1107/S1600536810001972/hg2630Isup2.hkl

Additional supplementary materials:  crystallographic information; 3D view; checkCIF report

## Figures and Tables

**Table 1:** Hydrogen-bond geometry (Å, °)

*D*—H⋯*A*	*D*—H	H⋯*A*	*D*⋯*A*	*D*—H⋯*A*
N1—H1*A*⋯O3^i^	0.88	1.96	2.842 (2)	176
C15—H15*A*⋯O1^ii^	0.98	2.48	3.434 (3)	164

Symmetry codes: (i) 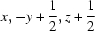; (ii) 
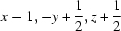
.

**Fig. 1.**
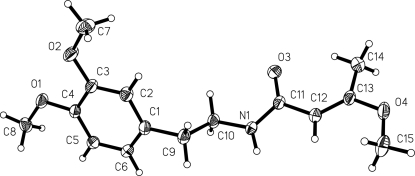
The molecular structure of (I), with atom labels and 40% probability displacement ellipsoids for non-H atoms.

## supplementary crystallographic information

### Comment

The title compound (*E*)—*N*-(3,4-dimethoxyphenethyl)-3-methoxybut-2-enamide was an important intermediate to the 3, 4-dihydroisoquinoline and some other heterocyclic compounds (Bernhard & Snieckus, 1971; Ma *et al.*, 2006). In this paper, we use 3,4-dimethoxyphenethylamine and 3-methoxy-2-butenoyl chloride to synthesize the title compound and report its crystal structure here.

The title compound C_15_H_21_NO_4_(Fig. 1), all bond lengths in the molecular are normal (Allen *et al.*, 1987). The intermolecular N—H···O hydrogen bonds [N···O 2.842 (2) Å] link the molecules related by translation along *c* axis into hydrogen-bonded chains.

### Experimental

3,4-dimethoxyphenethylamine (20 mmol) was solved in CH_2_Cl_2_, Et_3_N (30 mmol) was added, then 3-methoxy-2-butenoyl chloride (20 mmol) was added during 30 min at 273 K, after react 2 h at room temperature, the solution was washed with water, the organic layer was separated, dried with Na_2_SO_4_, evaporated to obtain the primary product, the pure product was isolated by recrystallization from ethyl acetate. (4.74 g, 84.9%). Single crystals suitable for X-ray measurements were obtained by recrystallization from ethyl acetate at room temperature.

### Refinement

H atoms were positioned geometrically and refined using a riding model, with C—H = 0.93–0.97 Å and N—H = 0.86 Å; with *U*_iso_(H) = 1.2 times *U*_eq_(C, N) and 1.5 times *U*_eq_(C)for methyl H atoms.

### Crystal data

**Table:**

C_15_H_21_NO_4_	*F*(000) = 600
*M**_r_* = 279.33	*D*_x_ = 1.256 Mg m^−^^3^
Monoclinic, *P*2_1_/*c*	Mo *K*α radiation, λ = 0.71073 Å
Hall symbol: -P 2ybc	Cell parameters from 4588 reflections
*a* = 12.509 (3) Å	θ = 1.4–27.5°
*b* = 14.930 (3) Å	µ = 0.09 mm^−^^1^
*c* = 8.2998 (17) Å	*T* = 173 K
β = 107.59 (3)°	Rod, colorless
*V* = 1477.5 (5) Å^3^	0.30 × 0.20 × 0.15 mm
*Z* = 4	

### Data collection

**Table:**

Rigaku Mercury CCD/AFC diffractometer	2591 independent reflections
Radiation source: Sealed Tube	2435 reflections with *I* > 2σ(*I*)
Graphite Monochromator	*R*_int_ = 0.049
φ and ω scans	θ_max_ = 25.0°, θ_min_ = 2.7°
Absorption correction: multi-scan (*CrystalClear*; Rigaku, 2007)	*h* = −14→14
*T*_min_ = 0.973, *T*_max_ = 0.987	*k* = −17→17
10754 measured reflections	*l* = −9→8

### Refinement

**Table:**

Refinement on *F*^2^	Primary atom site location: structure-invariant direct methods
Least-squares matrix: full	Secondary atom site location: difference Fourier map
*R*[*F*^2^ > 2σ(*F*^2^)] = 0.059	Hydrogen site location: inferred from neighbouring sites
*wR*(*F*^2^) = 0.133	H-atom parameters constrained
*S* = 1.18	*w* = 1/[σ^2^(*F*_o_^2^) + (0.045*P*)^2^ + 0.6856*P*] where *P* = (*F*_o_^2^ + 2*F*_c_^2^)/3
2591 reflections	(Δ/σ)_max_ < 0.001
181 parameters	Δρ_max_ = 0.16 e Å^−^^3^
0 restraints	Δρ_min_ = −0.18 e Å^−^^3^

### Special details

**Table:**

Geometry. All e.s.d.'s (except the e.s.d. in the dihedral angle between two l.s. planes) are estimated using the full covariance matrix. The cell e.s.d.'s are taken into account individually in the estimation of e.s.d.'s in distances, angles and torsion angles; correlations between e.s.d.'s in cell parameters are only used when they are defined by crystal symmetry. An approximate (isotropic) treatment of cell e.s.d.'s is used for estimating e.s.d.'s involving l.s. planes.
Refinement. Refinement of *F*^2^ against ALL reflections. The weighted *R*-factor *wR* and goodness of fit *S* are based on *F*^2^, conventional *R*-factors *R* are based on *F*, with *F* set to zero for negative *F*^2^. The threshold expression of *F*^2^ > σ(*F*^2^) is used only for calculating *R*-factors(gt) *etc*. and is not relevant to the choice of reflections for refinement. *R*-factors based on *F*^2^ are statistically about twice as large as those based on *F*, and *R*- factors based on ALL data will be even larger.

### Fractional atomic coordinates and isotropic or equivalent isotropic displacement parameters (Å^2^)

**Table:**

	*x*	*y*	*z*	*U*_iso_*/*U*_eq_	
O1	1.17428 (12)	0.48028 (10)	0.66017 (19)	0.0356 (4)	
O2	1.16017 (13)	0.30958 (10)	0.6857 (2)	0.0446 (4)	
O3	0.57963 (13)	0.16546 (10)	0.56367 (18)	0.0352 (4)	
O4	0.36035 (13)	0.06101 (10)	0.8340 (2)	0.0417 (4)	
N1	0.62813 (14)	0.26957 (12)	0.7715 (2)	0.0308 (4)	
H1A	0.6128	0.2920	0.8601	0.037*	
C1	0.91080 (17)	0.37551 (15)	0.8108 (3)	0.0303 (5)	
C2	0.99168 (17)	0.32078 (14)	0.7740 (3)	0.0320 (5)	
H2A	0.9871	0.2576	0.7838	0.038*	
C3	1.07767 (17)	0.35758 (14)	0.7239 (3)	0.0307 (5)	
C4	1.08529 (16)	0.45083 (14)	0.7088 (2)	0.0285 (5)	
C5	1.00533 (18)	0.50451 (14)	0.7431 (3)	0.0321 (5)	
H5A	1.0093	0.5677	0.7322	0.038*	
C6	0.91870 (18)	0.46703 (15)	0.7936 (3)	0.0331 (5)	
H6A	0.8642	0.5050	0.8167	0.040*	
C7	1.1604 (2)	0.21485 (16)	0.7102 (3)	0.0484 (6)	
H7A	1.2231	0.1881	0.6793	0.073*	
H7B	1.0896	0.1895	0.6390	0.073*	
H7C	1.1688	0.2019	0.8292	0.073*	
C8	1.1861 (2)	0.57474 (15)	0.6482 (3)	0.0379 (5)	
H8A	1.2524	0.5878	0.6126	0.057*	
H8B	1.1948	0.6022	0.7587	0.057*	
H8C	1.1192	0.5992	0.5650	0.057*	
C9	0.81715 (17)	0.33412 (16)	0.8642 (3)	0.0342 (5)	
H9A	0.7954	0.3757	0.9418	0.041*	
H9B	0.8445	0.2781	0.9271	0.041*	
C10	0.71449 (17)	0.31312 (15)	0.7155 (3)	0.0316 (5)	
H10A	0.6843	0.3693	0.6557	0.038*	
H10B	0.7364	0.2735	0.6351	0.038*	
C11	0.57000 (16)	0.19712 (14)	0.6969 (3)	0.0283 (5)	
C12	0.49832 (17)	0.15920 (14)	0.7913 (3)	0.0304 (5)	
H12A	0.5082	0.1820	0.9017	0.036*	
C13	0.42041 (17)	0.09551 (14)	0.7360 (3)	0.0321 (5)	
C14	0.3818 (2)	0.05392 (18)	0.5650 (3)	0.0500 (7)	
H14A	0.4248	0.0788	0.4944	0.075*	
H14B	0.3019	0.0667	0.5129	0.075*	
H14C	0.3933	−0.0110	0.5751	0.075*	
C15	0.3837 (2)	0.09322 (19)	1.0043 (3)	0.0500 (7)	
H15A	0.3349	0.0627	1.0594	0.075*	
H15B	0.3700	0.1579	1.0026	0.075*	
H15C	0.4623	0.0811	1.0669	0.075*	

### Atomic displacement parameters (Å^2^)

**Table:**

	*U*^11^	*U*^22^	*U*^33^	*U*^12^	*U*^13^	*U*^23^
O1	0.0300 (8)	0.0331 (8)	0.0494 (9)	−0.0036 (6)	0.0206 (7)	0.0028 (7)
O2	0.0372 (9)	0.0326 (9)	0.0727 (12)	0.0074 (7)	0.0298 (8)	0.0031 (8)
O3	0.0398 (9)	0.0370 (9)	0.0334 (9)	−0.0040 (7)	0.0180 (7)	−0.0029 (7)
O4	0.0338 (9)	0.0372 (9)	0.0607 (11)	−0.0059 (7)	0.0242 (8)	0.0051 (8)
N1	0.0318 (10)	0.0340 (10)	0.0330 (9)	−0.0074 (8)	0.0193 (8)	−0.0034 (8)
C1	0.0255 (11)	0.0361 (12)	0.0296 (11)	−0.0041 (9)	0.0087 (8)	−0.0002 (9)
C2	0.0300 (11)	0.0301 (12)	0.0365 (12)	0.0005 (9)	0.0109 (9)	0.0042 (9)
C3	0.0244 (10)	0.0324 (12)	0.0369 (12)	0.0027 (9)	0.0116 (9)	0.0016 (9)
C4	0.0230 (10)	0.0332 (11)	0.0301 (11)	−0.0028 (8)	0.0092 (8)	0.0003 (9)
C5	0.0319 (11)	0.0270 (11)	0.0399 (12)	−0.0020 (9)	0.0147 (9)	−0.0024 (9)
C6	0.0291 (11)	0.0347 (12)	0.0401 (12)	0.0000 (9)	0.0173 (9)	−0.0043 (9)
C7	0.0537 (16)	0.0342 (13)	0.0615 (16)	0.0135 (11)	0.0237 (13)	0.0057 (12)
C8	0.0401 (13)	0.0358 (13)	0.0407 (13)	−0.0122 (10)	0.0165 (10)	−0.0027 (10)
C9	0.0303 (12)	0.0414 (13)	0.0341 (12)	−0.0049 (10)	0.0144 (9)	0.0021 (10)
C10	0.0304 (11)	0.0358 (12)	0.0320 (12)	−0.0059 (9)	0.0147 (9)	0.0019 (9)
C11	0.0240 (10)	0.0298 (11)	0.0331 (12)	0.0011 (9)	0.0115 (9)	0.0029 (9)
C12	0.0294 (11)	0.0322 (12)	0.0318 (11)	−0.0025 (9)	0.0125 (9)	0.0018 (9)
C13	0.0244 (10)	0.0278 (11)	0.0476 (13)	0.0024 (9)	0.0164 (9)	0.0042 (9)
C14	0.0434 (14)	0.0491 (15)	0.0623 (17)	−0.0151 (12)	0.0235 (12)	−0.0199 (13)
C15	0.0445 (15)	0.0637 (17)	0.0492 (15)	−0.0082 (12)	0.0252 (12)	0.0135 (13)

### Geometric parameters (Å, °)

**Table:**

O1—C4	1.366 (2)	C7—H7B	0.9800
O1—C8	1.425 (3)	C7—H7C	0.9800
O2—C3	1.370 (2)	C8—H8A	0.9800
O2—C7	1.429 (3)	C8—H8B	0.9800
O3—C11	1.241 (2)	C8—H8C	0.9800
O4—C13	1.364 (2)	C9—C10	1.521 (3)
O4—C15	1.436 (3)	C9—H9A	0.9900
N1—C11	1.345 (3)	C9—H9B	0.9900
N1—C10	1.453 (2)	C10—H10A	0.9900
N1—H1A	0.8800	C10—H10B	0.9900
C1—C6	1.380 (3)	C11—C12	1.471 (3)
C1—C2	1.404 (3)	C12—C13	1.339 (3)
C1—C9	1.505 (3)	C12—H12A	0.9500
C2—C3	1.379 (3)	C13—C14	1.490 (3)
C2—H2A	0.9500	C14—H14A	0.9800
C3—C4	1.404 (3)	C14—H14B	0.9800
C4—C5	1.377 (3)	C14—H14C	0.9800
C5—C6	1.392 (3)	C15—H15A	0.9800
C5—H5A	0.9500	C15—H15B	0.9800
C6—H6A	0.9500	C15—H15C	0.9800
C7—H7A	0.9800		
			
C4—O1—C8	116.74 (16)	H8B—C8—H8C	109.5
C3—O2—C7	117.00 (18)	C1—C9—C10	112.77 (17)
C13—O4—C15	118.37 (18)	C1—C9—H9A	109.0
C11—N1—C10	124.27 (17)	C10—C9—H9A	109.0
C11—N1—H1A	117.9	C1—C9—H9B	109.0
C10—N1—H1A	117.9	C10—C9—H9B	109.0
C6—C1—C2	118.36 (19)	H9A—C9—H9B	107.8
C6—C1—C9	121.55 (19)	N1—C10—C9	111.08 (17)
C2—C1—C9	120.1 (2)	N1—C10—H10A	109.4
C3—C2—C1	120.8 (2)	C9—C10—H10A	109.4
C3—C2—H2A	119.6	N1—C10—H10B	109.4
C1—C2—H2A	119.6	C9—C10—H10B	109.4
O2—C3—C2	124.9 (2)	H10A—C10—H10B	108.0
O2—C3—C4	114.96 (18)	O3—C11—N1	122.13 (18)
C2—C3—C4	120.15 (19)	O3—C11—C12	124.53 (19)
O1—C4—C5	125.55 (19)	N1—C11—C12	113.31 (18)
O1—C4—C3	115.39 (18)	C13—C12—C11	126.0 (2)
C5—C4—C3	119.06 (19)	C13—C12—H12A	117.0
C4—C5—C6	120.6 (2)	C11—C12—H12A	117.0
C4—C5—H5A	119.7	C12—C13—O4	122.6 (2)
C6—C5—H5A	119.7	C12—C13—C14	128.0 (2)
C1—C6—C5	121.0 (2)	O4—C13—C14	109.43 (19)
C1—C6—H6A	119.5	C13—C14—H14A	109.5
C5—C6—H6A	119.5	C13—C14—H14B	109.5
O2—C7—H7A	109.5	H14A—C14—H14B	109.5
O2—C7—H7B	109.5	C13—C14—H14C	109.5
H7A—C7—H7B	109.5	H14A—C14—H14C	109.5
O2—C7—H7C	109.5	H14B—C14—H14C	109.5
H7A—C7—H7C	109.5	O4—C15—H15A	109.5
H7B—C7—H7C	109.5	O4—C15—H15B	109.5
O1—C8—H8A	109.5	H15A—C15—H15B	109.5
O1—C8—H8B	109.5	O4—C15—H15C	109.5
H8A—C8—H8B	109.5	H15A—C15—H15C	109.5
O1—C8—H8C	109.5	H15B—C15—H15C	109.5
H8A—C8—H8C	109.5		
			
C6—C1—C2—C3	0.7 (3)	C9—C1—C6—C5	−179.60 (19)
C9—C1—C2—C3	179.61 (19)	C4—C5—C6—C1	0.1 (3)
C7—O2—C3—C2	−4.2 (3)	C6—C1—C9—C10	89.1 (3)
C7—O2—C3—C4	175.8 (2)	C2—C1—C9—C10	−89.8 (2)
C1—C2—C3—O2	179.9 (2)	C11—N1—C10—C9	−134.8 (2)
C1—C2—C3—C4	−0.1 (3)	C1—C9—C10—N1	177.36 (18)
C8—O1—C4—C5	1.6 (3)	C10—N1—C11—O3	−5.9 (3)
C8—O1—C4—C3	−178.34 (18)	C10—N1—C11—C12	172.39 (18)
O2—C3—C4—O1	−0.7 (3)	O3—C11—C12—C13	−11.6 (3)
C2—C3—C4—O1	179.31 (18)	N1—C11—C12—C13	170.1 (2)
O2—C3—C4—C5	179.38 (19)	C11—C12—C13—O4	177.17 (19)
C2—C3—C4—C5	−0.6 (3)	C11—C12—C13—C14	−5.3 (4)
O1—C4—C5—C6	−179.30 (19)	C15—O4—C13—C12	−1.4 (3)
C3—C4—C5—C6	0.6 (3)	C15—O4—C13—C14	−179.3 (2)
C2—C1—C6—C5	−0.7 (3)		

### Hydrogen-bond geometry (Å, °)

**Table:**

*D*—H···*A*	*D*—H	H···*A*	*D*···*A*	*D*—H···*A*
N1—H1A···O3^i^	0.88	1.96	2.842 (2)	176
C15—H15A···O1^ii^	0.98	2.48	3.434 (3)	164

Symmetry codes: (i) *x*, −*y*+1/2, *z*+1/2; (ii) *x*−1, −*y*+1/2, *z*+1/2.
